# Abnormal intrahemispheric and interhemispheric dynamic functional connectivity density in male alcohol use disorder

**DOI:** 10.3389/fpsyt.2025.1531905

**Published:** 2025-06-27

**Authors:** Bohui Mei, Yarui Wei, Longyao Ma, Qiuying Tao, Jinghan Dang, Jieping Sun, Mengzhe Zhang, Yong Zhang, Jingliang Cheng

**Affiliations:** Department of Magnetic Resonance Imaging, The First Affiliated Hospital of Zhengzhou University, Zhengzhou, China

**Keywords:** alcohol use disorder, dynamic functional connectivity density, intrahemisphere connections, interhemisphere connections, resting-state functional magnetic resonance imaging

## Abstract

**Background:**

Previous studies have demonstrated abnormal static intrahemispheric and interhemispheric functional connectivity between different brain regions in patients with alcohol use disorder (AUD). However, brain activity is highly dynamic.

**Methods:**

To address this, we analyzed the dynamic changes in intrahemispheric and interhemispheric connectivity patterns from 55 AUD patients and 32 healthy controls. The whole-brain functional connectivity was decomposed into ipsilateral and contralateral components, and the voxel-wise intrahemispheric and interhemispheric dynamic functional connectivity density (dFCD) was calculated using a sliding window analysis. At the same time, the relationship between dFCD values in abnormal brain regions and clinical variables was conducted.

**Results:**

Our findings revealed that, compared to the HCs, AUD patients exhibited abnormal global, interhemispheric and intrahemispheric dFCD in the caudate, insula, parietal lobe, and occipital lobe. Furthermore, the dFCD values of these abnormal brain regions correlated with the average alcohol consumption and the severity of alcohol addiction in the AUD group.

**Conclusions:**

The results indicate that brain regions associated with the salience network, default mode network, and visual network exhibited intrahemispheric and interhemispheric abnormal functional connectivity. This study underscores that dynamic metrics can provide overlapping or complementary information alongside static metrics, contributing to a more comprehensive understanding of neural activity in AUD.

## 1 Introduction

Alcohol Use Disorder (AUD) is a psychiatric condition marked by an inability to control alcohol intake and a continued pursuit of drinking despite negative repercussions (1). According to a report released by the World Health Organization (WHO), more than 4% of the global population had an alcohol use disorder, and nearly 2.6 million people died due to the hazardous use of alcohol in 2019 (2). The detrimental consumption of alcohol is one of the primary risk factors jeopardizing global health, elevating the incidence of mental and behavioral disorders, infectious diseases, and injuries (3, 4), thereby imposing significant social and economic costs (5).

Recent advancements in neuroimaging technologies and sophisticated analysis techniques have made it possible to study the living human brain non-invasively. Functional magnetic resonance imaging (fMRI) can be employed to identify hemodynamic responses caused by neuronal activity in certain brain regions during task-based or resting states (6). This advancement presents a novel approach to the examination of resting-state functional connectivity (FC) and furnishes critical insights for assessing the integration of functional neuronal networks *in vivo* (7, 8). Numerous researchers have identified substantial alterations in the functional connectivity patterns of patients with AUD. Vergara, Liu (9) demonstrated that the precuneus, postcentral gyrus, insula, and visual cortex were the primary brain regions exhibiting diminished resting-state functional network connectivity in alcohol consumers. It has been reported that alcohol consumption adversely affects cognitive and executive functions (10), and certain research demonstrate that diminished connectivity within the executive control network correlates with high-risk alcohol use (11). Conversely, Han, Keedy (12) discovered that heavy alcohol use significantly enhances connections associated with the reward system, sensorimotor function, and cognitive control function. These inconsistent findings may be partly attributed to differences in methodological choices. For example, region of interest (ROI)-based methods rely on the selection of *a priori* seed points and may miss information about networks that are not predefined (13), while independent component analysis (ICA), although a data-driven method, still requires an artificially determined number of components and may lead to heterogeneity in network extraction due to different component selection strategies (14). These limitations may hinder the systematic exploration of whole-brain connectivity patterns.

To mitigate these restrictions, novel methodologies such as functional connectivity density (FCD) have been proposed. FCD is a data-driven method rooted in graph theory, quantifying the connections between voxels throughout the global, interhemispheric, or intrahemispheric brain. This method facilitates the identification of highly interconnected central distributions inside the network without relying on predefined seeds or components (15). In addition, previous studies have demonstrated widespread damage to the white matter microstructure in AUD, affecting vast areas including the genu and body of the corpus callosum, along with other commissural fibers (16–18). Such structural degradation hinders the efficiency of signal transmission across hemispheres (19). Moreover, Jansen, van Wingen (20) indicated that the functional connectivity of the cognitive control network between the left and right hemispheres in AUD is elevated compared to healthy controls. These findings collectively suggest that irregularities in anatomical or functional connection between hemispheres could disrupt functional interactions within the brain, ultimately undermining brain integrity in AUD. Consequently, it is imperative to examine the atypical functional connectivity within both interhemispheric and intrahemispheric brain networks in AUD with greater precision and comprehensiveness.

The aforementioned studies on AUD are based on the underlying assumption of spatial-temporal stability of fMRI data, which fails to adequately represent transitory fluctuations in spontaneous brain activity. A burgeoning corpus of research indicates that resting-state dynamics may be more pronounced when brain activity is unrestrained (21, 22). The dynamic functional connectivity density (dFCD) method has shown promise in various disorders, including major depression disorder (MDD) (23), generalized anxiety disorder (GAD) (24), and chronic smoking addiction (25). Additionally, the temporal variability of interhemispheric and intrahemispheric FCD has been explored in autism spectrum disorders (ASD) (26) and schizophrenia (27). It has been well established that integrating static and dynamic approaches can reveal fresh insights into aberrant functional connections in impaired brain areas and bilateral hemispheres in patients with diverse disorders. Nonetheless, to the best of our knowledge, it remains unclear whether AUD exhibit changes in the interhemispheric and intrahemispheric dFCD pattern.

This work will analyze the variation of dFCD both interhemispherically and intrahemispherically, utilizing the sliding window approach and referencing previous static research (28). This approach allows us to assess the time variability of brain activity and to identify the principal hub links in cerebral hemisphere network distributions. Additionally, we will explore the correlation between dFCD in abnormal brain regions and clinical variables.

## 2 Methods

### 2.1 Subjects

This study utilized the identical dataset as the prior research, comprising 32 age- and sex-matched healthy controls (HCs). AUD patients were recruited from inpatient wards, while participants in the HC group were volunteers from the local community. All subjects were male. Previous research indicate a substantial correlation between body mass index (BMI), smoking, and alcohol consumption (29, 30); therefore, we documented the BMI and smoking status of all participants. In addition, we assessed each subject’s duration of alcohol drinking, alcohol by volume, weekly frequency of drinking, the amount of alcohol drinking, and mean amount of pure alcohol per week (daily consumption × weekly frequency × consumption × 0.8). The subjects in the HC group did not consume alcohol. All subjects were male. The inclusion criteria for the AUD group were as follows: (1) fulfillment of DSM-V criteria (at least two of the 11 potential AUD symptoms); (2) aged ranging from 18 to 65 years; (3) average consumption exceeding 14 units of alcohol per week (31); (4) no current use of psychotropic drugs at the time of recruitment or off anti-addiction medication for at least 12 hours; (5) no family history of alcohol dependence; and (6) right­handedness. The exclusion criteria for both the AUD and HC groups were: (1) a history of psychiatric, neurological, or physical disorders; (2) the presence of contraindications for magnetic resonance imaging (MRI); and (3) taking psychotropic or other medications at the time of recruitment.

We used Montreal cognitive assessment (MoCA) to measure whether individuals with AUD had cognitive impairment (32). Further, all patients completed the following assessments to evaluate the severity of AUD: the alcohol dependence scale (ADS), the alcohol use disorder identification test (AUDIT), the cutdown, annoyed, guilty, eye-opener (CAGE) scale, the clinical institute withdrawal assessment-advanced revised (CIWA-Ar) scale, the Michigan alcoholism screening test (MAST), the obsessive compulsive drinking scale (OCDS), and the visual analogue scale (VAS). All subjects provided informed consent, and this study was approved by the Ethics Committee of the First Affiliated Hospital of Zhengzhou University.

### 2.2 Data collection

All MRI images were acquired using a 3.0T MRI scanner (MAGNETOM Prisma, SIEMENS, Germany) with a 64-channel receiver array head coil. Subjects were instructed to close their eyes, remain still and not sleep during the scan use foam fillers and earplugs to minimize head movement and scanner noise. And the subjects were asked again at the end of the scan to ensure that they had not slept. The simultaneous multi-slice imaging technique was employed to obtain functional images using a gradient-echo echo-planar imaging (GRE-EPI) sequence. The acquisition parameters are as follows: repetition time (TR)/echo time (TE) = 1,000/30 ms; slice number = 52; slice thickness = 2.2 mm; slice gap = 0.4 mm; flip angle = 70°; field of view (FOV) = 17.6 × 17.6 cm²; number of averages = 1; matrix size = 64 × 64; voxel size = 2.75 × 2.75 × 2.2 mm³; slice acceleration factor = 4; integrated parallel acquisition technology (iPAT) acceleration factor = 2; and acquisition bandwidth = 1,750 Hz/Px. A total of 400 volumes were acquired with a scanning time of 6.67 minutes.

### 2.3 Data processing

Functional images underwent preprocessing using Data Processing Assistant for Resting-State fMRI (DPARSF) software, based on Statistical Parametric Mapping (SPM12, http://www.fil.ion.ucl.ac.uk/spm) and MATLAB 2018b (MathWorks, Natick, MA USA). The primary preprocessing stages comprised the elimination of the initial 10 volumes to reduce early signal instability, slice timing correction, and realignment. Data on subjects with translational or rotational head movement above 3 mm or 3° during scanning were excluded. Next, the remaining data were normalized to the Montreal Neurological Institute (MNI) space with a resampling voxel size of 3 × 3 × 3 mm³, detrended, and bandpass filtering (0.01-0.1Hz). Then, image volumes with framewise displacement (FD) >0.5mm underwent scrubbing using cubic spline interpolation to further exclude motion artifacts (33). Linear regression was ultimately employed to eliminate the influence of nuisance factors, such as the Friston 24 head motion parameters, as well as signals from white matter and cerebrospinal fluid (34).

### 2.4 dFCD calculation and temporal variability

The sliding window approach was employed to compute global, contralateral, and ipsilateral FCD time variability. The window length is a crucial parameter in the calculation of resting-state dynamics. A brief window length heightens the likelihood of incorporating spurious fluctuations, whereas extended window lengths may conceal the examination of lower-frequency fluctuations pertinent to the signal (35). Consequently, drawing from prior research (21, 36, 37), we chose a sliding window ranging from 10 to 180 seconds, with a window length of 100 TRs and a step size of 2 TR to calculate the temporal variation of FCD. The ensuing calculations resembled those of our prior investigation on static FCD (28). In simple terms, we first calculated the global FCD for each window, representing the mean number of functional connections between each voxel (seed) and other voxels (target voxels) over the entire brain. The global FCD is limited to voxels within the gray matter template. Pearson correlation was used to evaluate the connectivity between two voxels, with the threshold for the correlation coefficient set at p < 0.05 without correction. Connectivity was deemed existent when the correlation coefficient between two voxels surpassed the threshold. Subsequently, based on the seed voxel and the relative position of the target voxel, the global FCD is partitioned into contralateral and ipsilateral FCD. The contralateral (interhemispheric) FCD of each voxel refers to the number of voxels with a correlation coefficient above the threshold in the contralateral hemisphere, while the ipsilateral (intrahemispheric) FCD of each voxel refers to the number of voxels with a correlation coefficient above the threshold in the same hemisphere. Additionally, to investigate the reproducibility of the results, we replicated our findings with different window lengths (60 and 160 TRs), correlation thresholds (p < 0.01 and p < 0.001), and moving step sizes (4 TRs). The temporal variability of global, contralateral, and ipsilateral dFCD was estimated by calculating the variance of the sliding window FCD. We then transformed the temporal fluctuations of each figure to a z-score matrix and applied isotropic Gaussian kernel smoothing (full width at half maximum (FWHM) = 6 mm).

### 2.5 Statistical analysis

Statistical analysis was performed based on IBM SPSS Statistics version 19.0. The normality of demographic, clinical and head motion data in each group was examined by the Shapiro-Wilk test. The two-sample t test was used to check for differences between groups in normally distributed data, while the Mann-Whitney test was used for differences between groups in non-normally distributed data. To further investigate changes in global, interhemispheric and intrahemispheric dFCD temporal variability, two-sample t tests were performed between the AUD and HC groups, with age, years of education and mean FD included as covariates. The findings presented in this study were adjusted for multiple comparisons (voxel-wise p < 0.001, cluster-level p < 0.05; Gaussian random field (GRF) correction). To evaluate the relationship between the abnormal FC patterns of AUD and clinical variables, the ROI were delineated as spheres with a radius of 6 mm centered on the brain regions that showed significant global, interhemispheric and intrahemispheric FCD differences between the two groups. The mean variance of dFCD for all ROIs was extracted, and the correlation with clinical markers was assessed (p < 0.05; two-tailed) to investigate potential associations.

## 3 Results

### 3.1 Clinical demographics

There were no significant differences in age (Z = -1.696, P = 0.09) or BMI (Z = -1.778, P = 0.076) between the AUD and HC groups. For detailed information, please refer to **Table 1**. We did not detect any significant differences between the groups in terms of mean FD (Z = −1.743, P = 0.081) or in the number of image volumes scrubbed (Z = −1.166, P = 0.247).

**Table 1:** The demographic and clinical data of AUD and HC.

	AUD (n = 55)	HC (n = 32)	*P* values
Age (years, SD)	45.56 (8.88)	42.47 (12.34)	0.090
BMI (kg/m^2^, SD)	25.37 (2.92)	26.31 (2.21)	0.076
Education (years, SD)	9.67(3.6)	10.74(3.56)	0.213
Smoking status (AUD: n = 49; HC = 17)			
Smoker	32	17	–
Non-smoker	17	–	–
Duration of alcohol drinking (years, n = 29, SD)	20.14 (9.89)	–	–
Alcohol by volume (%, n = 28, SD)	51.71 (1.08)	–	–
Frequency of alcohol drinking (days/week, n = 30, SD)	4.09 (1.91)	–	–
Amount of alcohol drinking (ml/day, n = 30, SD)	238.33 (108.82)	–	–
Mean amount of pure alcohol (g/week, n = 30, SD)	522.38 (333.83)	–	–
CAGE (n = 33, SD)	1.18 (0.98)	–	–
AUDIT (n = 34, SD)	19.97 (7.22)	–	–
CIWA-Ar (n = 29, SD)	9.34 (7.63)		
MAST (n = 31, SD)	15.00 (8.58)	–	–
ADS (n = 41, SD)	12.76 (6.66)	–	–
OCDS (n = 31, SD)	14.42 (9.95)	–	–
VAS (n = 29, SD)	2.29 (2)	–	–
MoCA (n = 29, SD)	28.24 (1.27)	–	–

ADS, alcohol dependence scale; AUD, alcohol use disorder; AUDIT, alcohol use disorder identification test; BMI, body mass index; CAGE, cutdown, annoyed, guilty, eye-opener; CIWA-Ar, Clinical Institute Withdrawal Assessment-advanced Revised; HC, healthy control; MAST, Michigan alcoholism screening test; MoCA, Montreal cognitive assessment; OCDS, obsessive compulsive drinking scale; VAS, visual analogue scale.

### 3.2 Dynamic FCD differences

**Figure 1:**
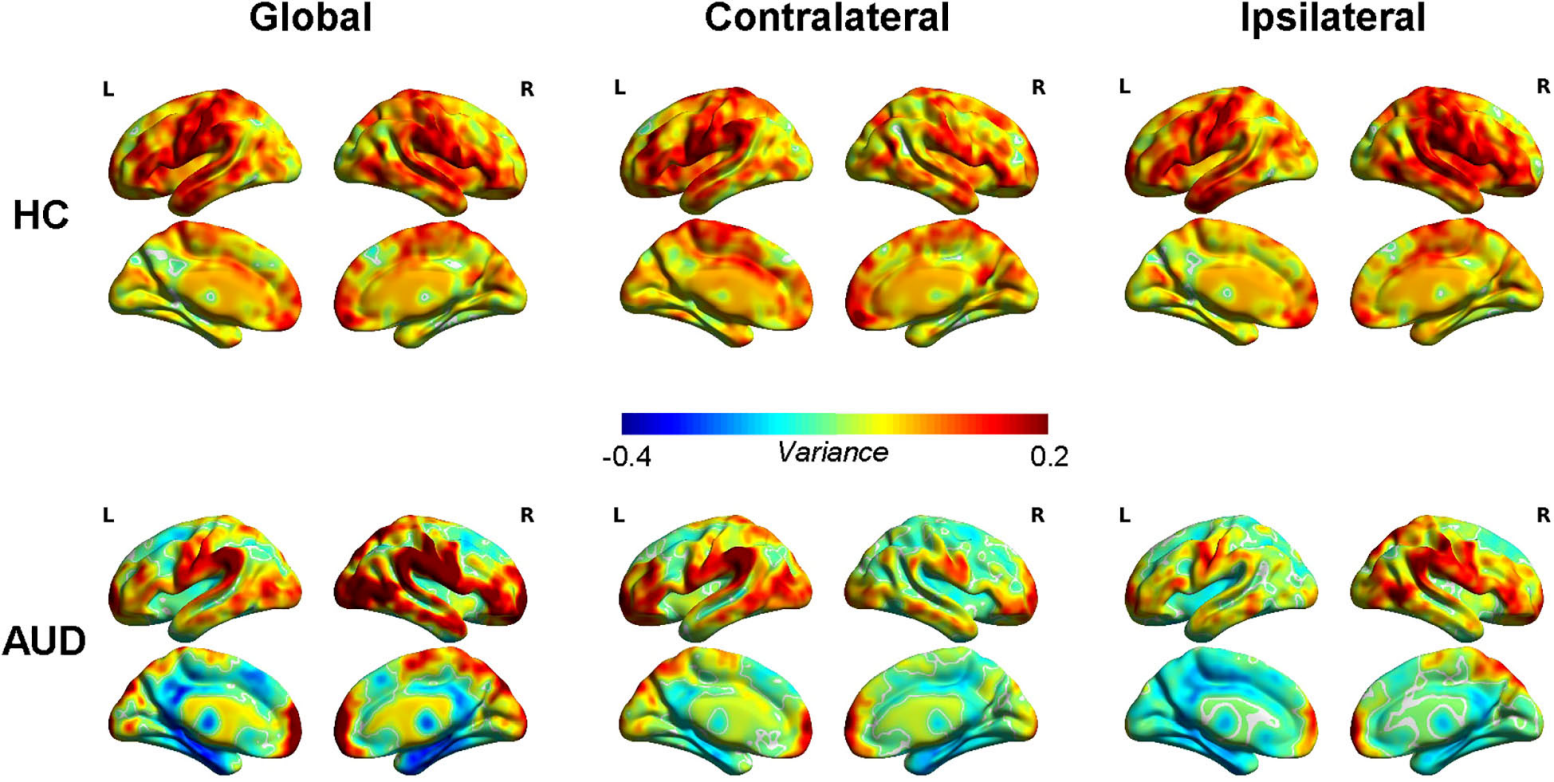
Average dynamic functional connectivity density (dFCD) variance for healthy controls (HCs) and alcohol use disorder (AUD).

The average global, contralateral, and ipsilateral dFCD variation maps for the two groups are presented in **Figure 1**. The HC group exhibited the greatest variability in dFCD within the bilateral anterior middle frontal gyrus (MFG), bilateral inferior frontal gyrus (IFG), bilateral temporal lobe, bilateral supramarginal gyrus, bilateral angular gyrus, bilateral precentral gyrus, left postcentral gyrus, and right insula. In contrast, the least variable dFCD was found in the bilateral thalamus, bilateral precuneus, right internal superior frontal gyrus (SFG), and left cuneus.

**Figure 2:**
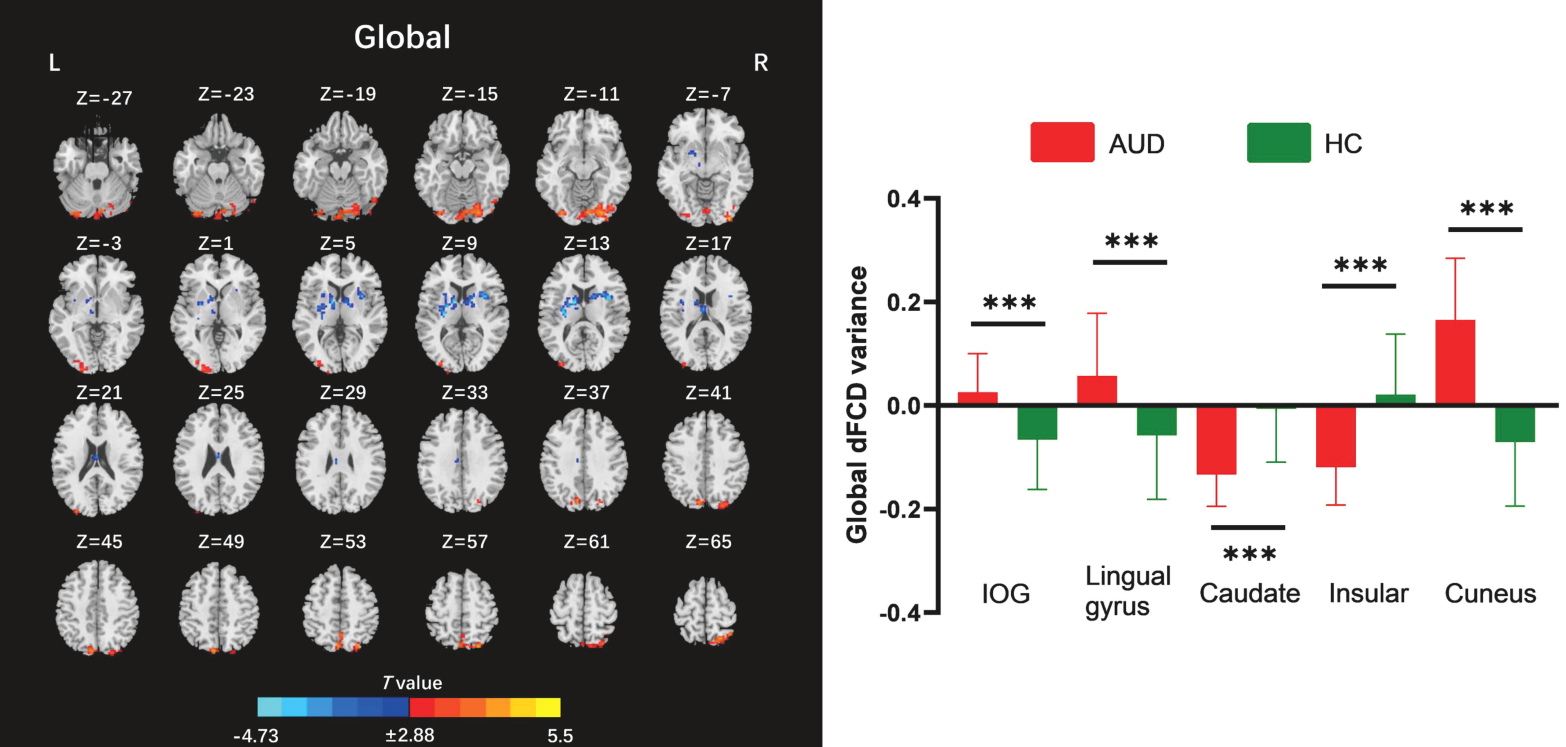
Between-group differences for global dynamic functional connectivity density (dFCD). AUD, alcohol use disorder; HC, healthy control; IOG, inferior occipital gyrus; L, left; R, right. ***p < 0.001.

**Figure 3:**
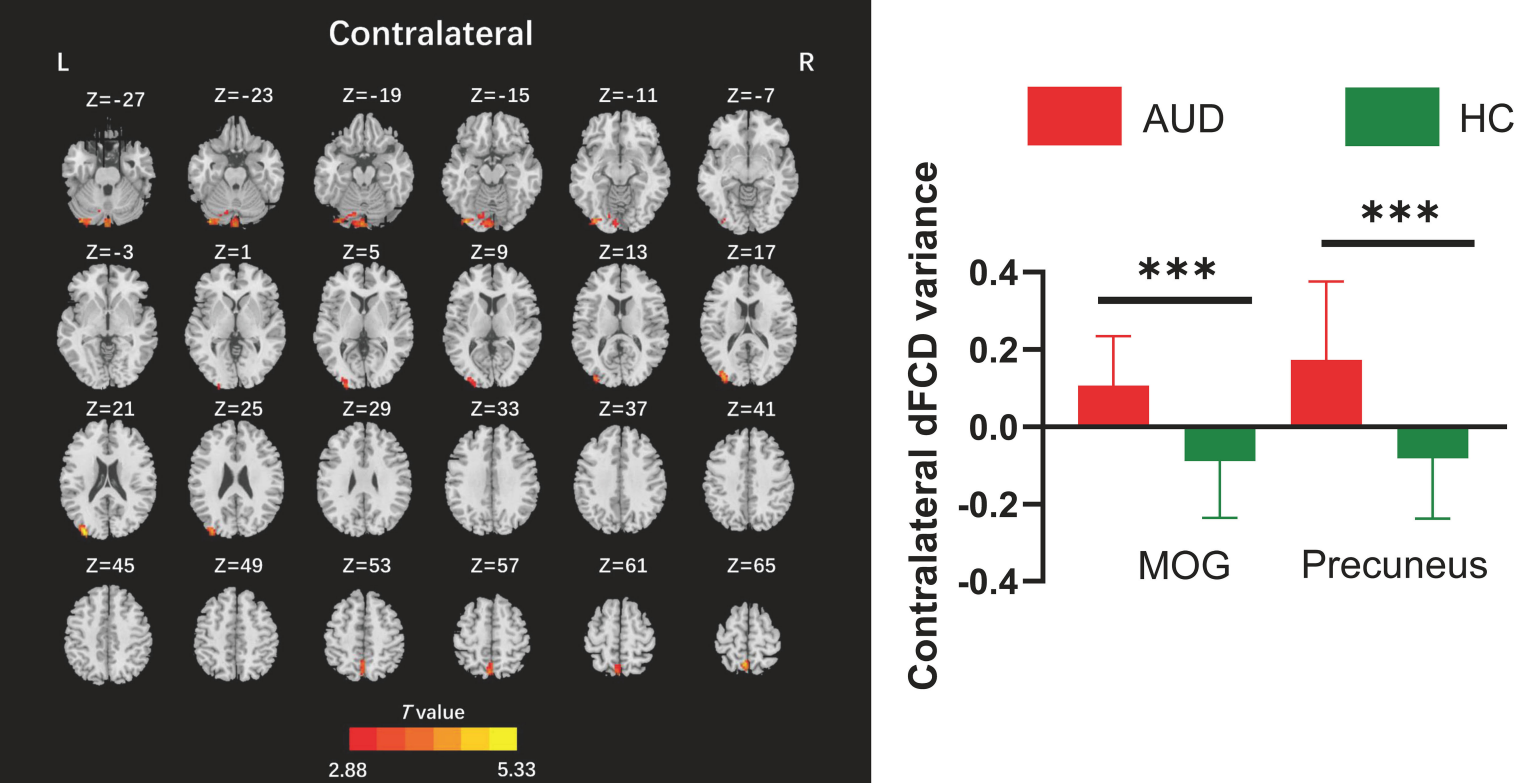
Between-group differences for contralateral dynamic functional connectivity density (dFCD). AUD, alcohol use disorder; HC, healthy control; L, left; MOG, middle occipital gyrus; R, right. ***p < 0.001.

**Figure 4:**
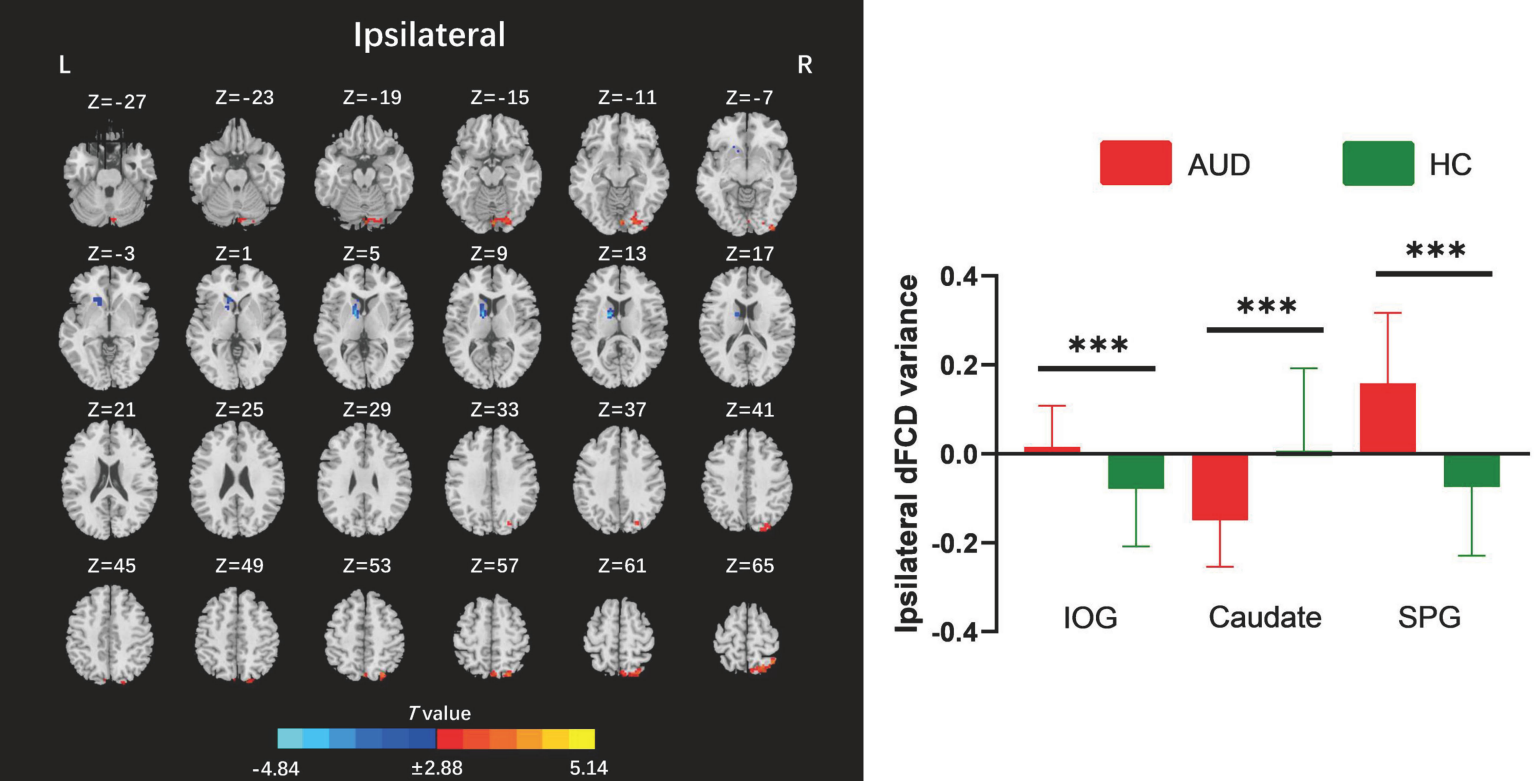
Between-group differences for ipsilateral dynamic functional connectivity density (dFCD). AUD, alcohol use disorder; HC, healthy control; IOG, inferior occipital gyrus; L, left; R, right; SPG, superior parietal gyrus. ***p < 0.001.

The two-sample t-test results indicated that, relative to the HC group, AUD patients demonstrated heightened average global dFCD variability in the right inferior occipital gyrus (IOG), left lingual gyrus, and left cuneus, while exhibiting diminished variability in the left caudate and right insula (**Table 2**; **Figure 2**). Compared to the HC group, the AUD group also showed increased interhemispheric dFCD variability in the left middle occipital gyrus (MOG) and left precuneus (**Table 2**; **Figure 3**). The variability of intrahemispheric dFCD exhibited a pattern analogous to that of global dFCD, characterized by higher intrahemispheric dFCD in the right IOG and right superior parietal gyrus (SPG), and decreased levels in the left caudate (**Table 2**; **Figure 4**).

**Table 2:** Between-group differences in the global, contralateral, and ipsilateral dFCD variances.

dFCD	Regions	Hemisphere	Cluster size (voxels)	Peak MNI coordinate			Peak *T* values
				X	Y	Z	
Global	IOG	R	384	36	-93	-9	4.87
	Lingual	L	199	-33	-87	-12	4.32
	Caudate	L	244	-12	6	12	-4.73
	Insula	R	107	36	6	12	-4.50
	Cuneus	L	284	-9	-78	39	5.50
Contralateral	MOG	L	105	-33	-90	21	5.34
	Precuneus	L	110	-3	-57	69	4.90
Ipsilateral	IOG	R	96	36	-93	-9	4.14
	Caudate	L	100	-12	2	14	-4.84
	SPG	R	197	12	-63	72	5.14

The statistically significant threshold was set at P < 0.001 (voxel-wise) and P < 0.05 (cluster-wise) and the minimum cluster size was 72 voxels following Gaussian random field (GRF) correction. dFCD, dynamic functional connectivity density; IOG, inferior occipital gyrus; L, left; MOG, middle occipital gyrus; R, right; SPG, superior parietal gyrus.

### 3.3 Result-Correlation analysis

**Figure 5:**
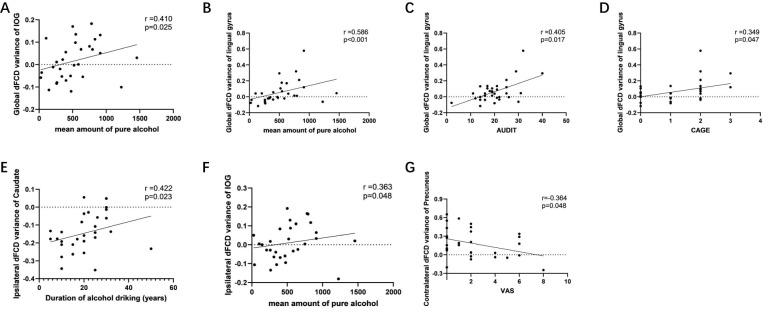
Associations between abnormal global, ipsilateral and contralateral dynamic functional connectivity density (dFCD) and the symptom severity of alcohol use disorder. Among the abnormal brain regions of AUD patients, **(a)** the global dFCD variance in the right IOG exhibited a positive correlation with the mean amount of pure alcohol; the global dFCD variance in the left lingual gyrus exhibited a positive correlation with **(b)** the mean amount of pure alcohol, **(c)** AUDIT (r=0.405, p=0.017), and **(d)** CAGE scores. **(e)** the variance of the variance of ipsilateral dFCD in the left caudate nucleus exhibited a positive correlation with the duration of alcohol drinking; **(f)** the ipsilateral dFCD in the right IOG demonstrated a positive correlation with the mean amount of pure alcohol; **(g)** the variance of contralateral dFCD in the left precuneus exhibited a negative correlation with VAS. AUDIT, alcohol use disorder identification test; CAGE, cutdown, annoyed, guilty, eye-opener; IOG, inferior occipital gyrus; VAS, visual analogue scale.

Among the abnormal brain regions of AUD patients, the global dFCD variance in the right IOG exhibited a positive correlation with the mean amount of pure alcohol (r=0.410, p=0.025) (**Figure 5A**). The global dFCD variance in the left lingual gyrus exhibited a positive correlation with the mean amount of pure alcohol (r=0.586, p=0.0007) (**Figure 5B**), AUDIT (r=0.405, p=0.017) (**Figure 5C**), and CAGE scores (r=0.349, p=0.047) (**Figure 5D**). The variance of the variance of ipsilateral dFCD in the left caudate nucleus exhibited a positive correlation with the duration of alcohol drinking (r=0.422, p=0.023) (**Figure 5E**), while ipsilateral dFCD in the right IOG demonstrated a positive correlation with the mean amount of pure alcohol (r=0.364, p=0.048) (**Figure 5F**). The variance of contralateral dFCD in the left precuneus exhibited a negative correlation with VAS (r=-0.364, p=0.048) (**Figure 5G**).

### 3.4 Validation analysis

In our study, we corroborated our findings by employing various window lengths (60 and 160 TRs), correlation thresholds (p <0.01 and p <0.001), and moving step sizes (4 TRs). The ultimate outcomes aligned with our primary dFCD findings (**Supplementary Figures S1-S3**). This adequately illustrates the stability and reproducibility of our findings.

## 4 Discussion

This study employs FCD and sliding window analysis to concurrently decompose global brain FCD into ipsilateral and contralateral components. This facilitates the analysis of interhemispheric and intrahemispheric FCD, uncovering the temporal anomaly patterns within the network in individuals with AUD. Relative to the HC group, the average global variation pattern in the AUD group resembled the intrahemispheric pattern, exhibiting an increase in the right IOG and decreases in the left caudate. Moreover, AUD patients had distinct augmentations in the left lingual gyrus and left cuneus, coupled with reductions in the right insula within the global variation pattern. Interhemispheric variation revealed enhancements in the left MOG and the left precuneus. These findings suggest that both interhemispheric and intrahemispheric dFCD can provide complementary information to static indices, aiding in a more comprehensive understanding of neural activity and functional biomarkers in AUD patients.

Our results exhibited a decrease in the mean global dFCD in the insula and caudate of the core regions of the salience network (SN). Disruption and disconnection of the SN with other networks, such as the default mode network (DMN), have been documented in numerous neuropsychiatric disorders (38), particularly in AUD. The SN is crucial for monitoring, integrating, and filtering pertinent events and information (39), encompassing cognition and motivation (40), and for controlling the transition between the DMN and executive control network (ECN) (41). Additional studies indicate that the right insula governs this transition; upon external stimulation, the brain shifts from the DMN, oriented towards internal processes, to the external perceptual awareness of the ECN (42, 43). Studies utilizing fMRI and arterial spin labeling (ASL) indicates reduced insula perfusion and diminished functional connectivity across the anterior cingulate cortex (ACC), insula, parietal lobe, and medial frontal regions in AUD (44). It has also been reported that acute alcohol intake impacts the functional connectivity between the insula and the frontoparietal control network, thereby influencing emotional expression (45). Our results corroborate prior studies, underscoring the insula as a pivotal center in the interplay of the SN, DMN, and ECN (46). These results suggest that the insula is crucial in the brain’s functional changes in AUD patients and may represent a promising therapeutic target for impulse control issues associated with alcohol addiction.

The precuneus, a part of the parietal lobule, serves as the functional core of the DMN (47) and is crucial for self-referential processing, vigilance, environmental monitoring (48), and the maintenance of cognition, emotion, and memory (49). Abnormal DMN patterns have been observed in populations with various substance use disorders (50). Our study shows that compared to HCs, AUD patients exhibit increased interhemispheric dFCD variability in the left precuneus and elevated intrahemispheric dFCD variability in the right SPG. This probably reflects impaired resting-state DMN connectivity in AUD patients, especially in the right hemisphere. Previous studies indicate that the non-dominant hemisphere (often the right) is integral to major cognitive functions, including visuospatial skills, social cognition, and socioemotional psychology (51–53). Hence, the abnormal connection of the DMN to the right hemisphere in AUD patients leads to dysfunctions in attention, cognition, and control, potentially fostering alcohol dependency. Moreover, variability in hyperconnectivity within the DMN may result in overthinking, causing patients to concentrate excessively on their internal experiences, engrossed in past or imagined addictive situations, thus exacerbating addiction (54). These patients exhibit increased vulnerability to cognitive deficits and psychiatric disorders, including depression (55). Thus, heightened variability in the DMN may elucidate a neurological basis for the comorbidity of AUD and depression, presenting prospective neural targets for the therapy of these comorbid conditions.

The visual network (VIN) constitutes a component of the sensory cortical system, encompassing regions such as the IOG, MOG, lingual gyrus, and cuneus. The occipital lobe, as the hub of the visual cortex, is accountable for an array of visual processes such as visual processing and memory encoding (56), and is also linked to executive function and attention (57). The lingual gyrus is an essential part of the occipital lobe, associated with the visual cortex (58), while the cuneus is thought to play a crucial role in visual information integration (59, 60). Our results reveal that these regions exhibit heightened global, interhemispheric and intrahemispheric dFCD variability, compared to HCs, congruent with our prior findings on static FCD (28). The resemblance between static and dynamic FCD indices may indicate a fundamental adjustment mechanism, serving as a compensatory reaction, wherein the visual network and overall brain connection in AUD patients are enhanced, but with heightened variability. Furthermore, correlation analyses indicated that the global and ipsilateral dFCD values of the IOG and lingual gyrus were positively correlated with the mean amount of pure alcohol and the intensity of addiction. These results validate that AUD patients often experience visual processing deficits, providing a theoretical foundation for future targeted treatments or preventive measures for AUD via visual stimuli interventions. Moreover, the realization of specific brain functions hinges on real-time information exchange within and between networks, as well as the reasonable allocation of different networks (61, 62), which may be the reason why the abnormal dFCD observed in a wide range of regions.

Notably, comparison of the present study with previous related static studies (28) revealed that although dynamic FCD focuses on temporal fluctuation properties while static FCD reflects steady-state connectivity strengths, the two approaches showed significant concordance in the pattern of abnormality in key brain regions, viz, decreased FCD in the left caudate and the right insula; visually related cortex such as the right IOG, left lingual gyrus, left cuneus, and sensory integration regions such as the right SPG, the left precuneus were increased in FCD. This cross-methodological consistency suggests that functional abnormalities in the above brain regions may be a core feature of AUD neuropathology, further supporting their potential as therapeutic targets for AUD. In addition, static analyses detected additional abnormal regions such as the thalamus and cingulate gyrus, which may reflect the cumulative effects of long-term alcohol exposure, and altered homeostatic connectivity in these regions may be progressively accentuated during the chronicity of the disease. The results of the two methods can be complementary (59) suggesting that combining the spatiotemporal multidimensional perspective can provide a more complete resolution of the neural mechanisms of AUD.

Several limitations of our research need to be acknowledged. Firstly, participants in the AUD group were sourced from hospital wards, signifying that the patients had consented to or were inclined to pursue therapy. Nonetheless, the majority of people with these conditions remain oblivious to their affliction or are reluctant to pursue treatment. Consequently, the samples in this investigation may not accurately represent all AUD patients, necessitating enhancements in future experimental design. Secondly, the sample size of our study is relatively insufficient, and the participants exhibit no symptoms of cognitive impairment, with the severity of symptoms being minimal, which may restrict the generalizability of our findings. Subsequent research should encompass a bigger participant pool and examine related cognitive problems more thoroughly to guarantee clinical repeatability. Thirdly, as a case-control cross-sectional study, it is challenging to determine the causal relationship between changes in FCD patterns and alcohol intake accurately. Longitudinal investigations are warranted. Fourthly, previous studies indicate that changes in brain function and structure associated with AUD vary by gender, race, and age (63). The subjects of this study were solely middle-aged Han Chinese men, thereby restricting the generalizability to other demographics, including women, teenagers, and smokers from various ethnicities. Lastly, as a single-center study with a small sample size, the findings necessitate validation through multicenter data. These findings warrant further confirmation in future studies.

## 5 Conclusion

Taken together, our work delineates specific patterns of global, interhemispheric, and intrahemispheric dFCD deficits in AUD patients, mainly focusing on the SN, DMN, and essential elements of the visual pathway. Notably, abnormal connectivity between the DMN and the right hemisphere may significantly contribute to cognitive dysfunction in AUD patients. This study maps the distribution of primary hub links within the hemispherical framework of aberrant brain networks in AUD patients, proposing that critical nodes, including the insula, cuneus, and precuneus, may serve as viable therapeutic targets. Additionally, it enhances the comprehension of the disease’s pathophysiology, suggesting that factors such as topological location and anatomical distance ought to be taken into account in forthcoming investigations of the aberrant brain network in AUD.

## Data Availability

The original contributions presented in the study are included in the article/**Supplementary Material**. Further inquiries can be directed to the corresponding authors.
