# Supplementary material for: Abnormal intrahemispheric and interhemispheric dynamic functional connectivity density in male alcohol use disorder
Source: Front Psychiatry. 2025 Jun 27;16:1531905. doi: 10.3389/fpsyt.2025.1531905 (PMC12245767; doi:10.3389/fpsyt.2025.1531905)
Supplement: Supplementary file 1 [file DataSheet1.docx]

***Supplementary Material***

**Methods and Results**

**Validation analysis**

In our study, we validated our results by using different window lengths (60 and 160 TRs), correlation thresholds (p <0.01 and p <0.001), and moving step sizes (4 TRs). The final results were consistent with our main dFCD results [Figure S1-S3]. This is sufficient to demonstrate the stability and reproducibility of our results.


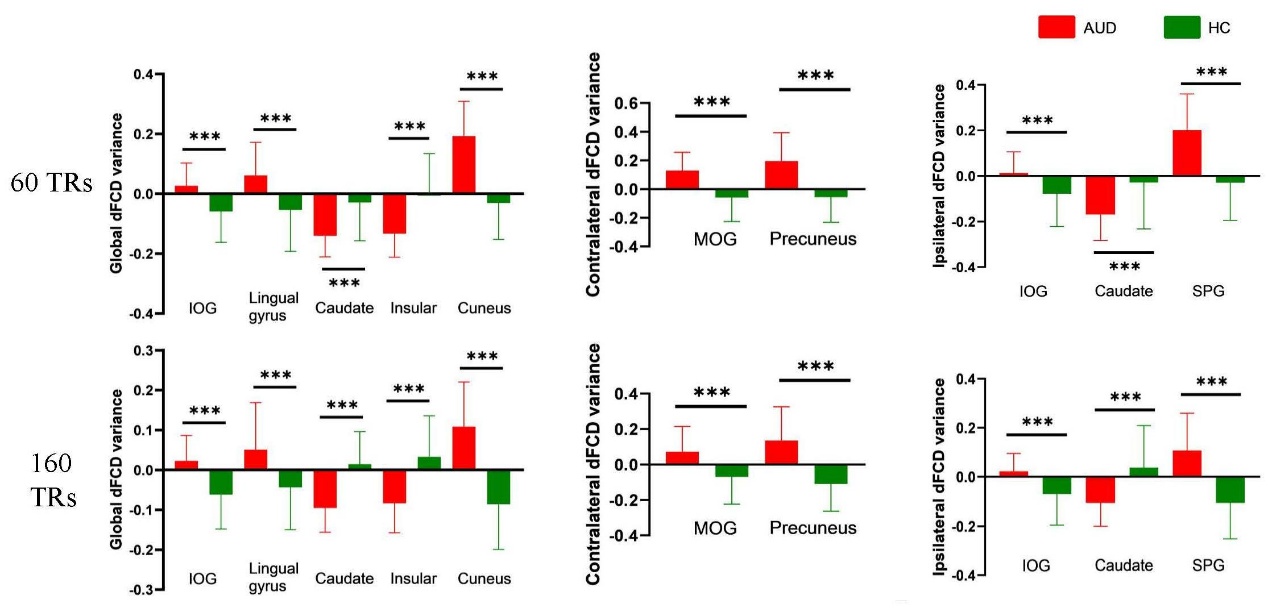


**Figure S1** Replication analyses of window lengths. AUD, alcohol use disorder; HC, healthy control; IOG, inferior occipital gyrus; L, left; MOG, middle occipital gyrus; R, right; SPG, superior parietal gyrus. ********p* < 0.001.


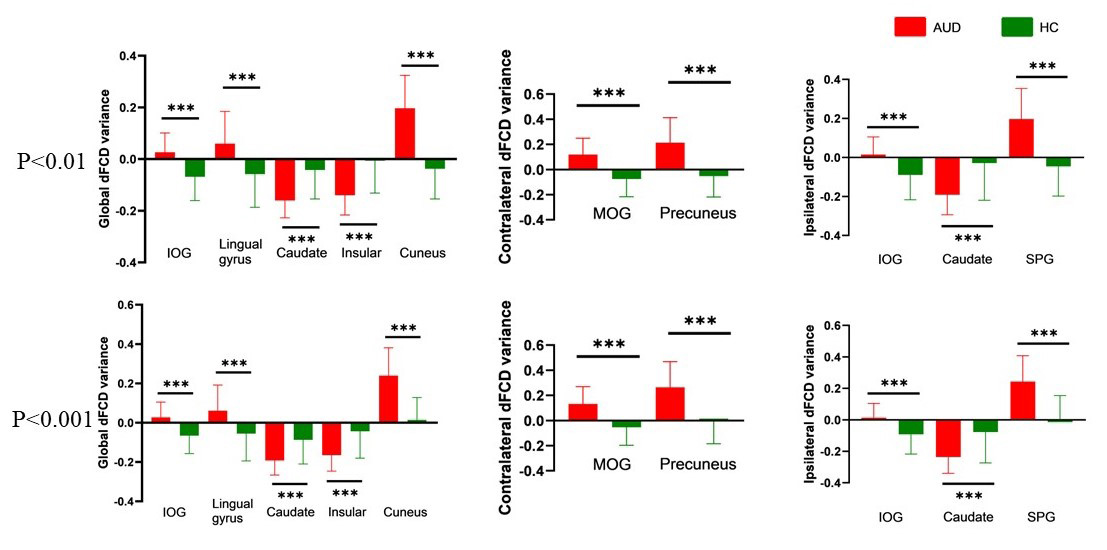


**Figure S2** Validation analyses of correlation thresholds. AUD, alcohol use disorder; HC, healthy control; IOG, inferior occipital gyrus; L, left; MOG, middle occipital gyrus; R, right; SPG, superior parietal gyrus. ***p < 0.001.


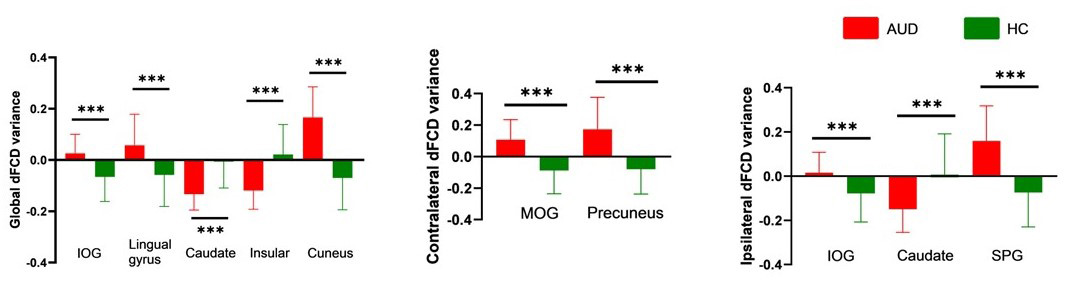


**Figure S3** Validation analyses of shifting step. AUD, alcohol use disorder; HC, healthy control; IOG, inferior occipital gyrus; L, left; MOG, middle occipital gyrus; R, right; SPG, superior parietal gyrus. ***p < 0.001.
